# A comparison of commercially available synthetic skin substitutes for surgical simulation training

**DOI:** 10.3205/zma001644

**Published:** 2023-09-15

**Authors:** Laura Awad, Benjamin J. Langridge, Faith H. K. Jeon, Edward Bollen, Peter E. M. Butler

**Affiliations:** 1 Royal Free Hospital, Charles Wolfson Center for Reconstructive Surgery, London, United Kingdom; 2 Royal Free Hospital, Department of Plastic Surgery, London, United Kingdom; 3 Royal Free Hospital, Department of Plastic and Reconstructive Surgery, London, United Kingdom

**Keywords:** skin pads, skin substitutes, simulation, surgical skills, remote learning

## Abstract

**Objective:**

Simulation training provides an important opportunity to accelerate surgical skills acquisition whilst safeguarding patients. This study compares the suitability of different synthetic skin substitutes for use in surgical simulation training.

**Design:**

Data was collected for eight commercially available synthetic skin substitutes and included cost, delivery time, subjective assessment of fidelity by surgeons and trainees, and objective comparison with the biomechanics of human skin was made through cutometry and durometry measurements. Cutometry and durometry data was collected from three healthy adults from the forearm, forehead and back, with measurements being repeated in triplicate. Subjective assessment of skin pad quality was collected using an 8-criteria questionnaire, graded using a 5-point Likert scale for fidelity to normal skin.

**Results:**

The questionnaire assessment was completed by 30 trainees and practitioners. Overall, felt pads received the poorest outcomes in all criteria; cutometry and durometry results demonstrate poor similarity to skin, and felt received the lowest scores in the questionnaire, although the cheapest. Foam dressings were similar in both cutometric and durometric properties to skin of the face, back and arm. Clinical outcomes of foam dressings were similar to the most expensive commercial skin pad.

**Conclusions:**

Bilaminar foam-based dressings provide a low cost, high fidelity non-biological simulation of skin for surgical training, which is non-inferior to more expensive specifically designed products. Many products designed to act as skin substitutes for surgical simulation fail to adequately replicate the anatomical and mechanical properties of skin.

## 1. Introduction

Providing opportunities for surgical training outside of the operating theatre is essential for effective training of junior surgeons. With reduced theatre activity, a high number of trainees, and theatres presenting high risk environments, surgical skills training in a simulated setting can provide a solution to difficulties encountered in skill development, and improve the safety of this learning process for both patients and trainees [1]. Surgical simulation as a training modality is increasing in popularity in many educational centres, with the use of both low fidelity low cost, and high fidelity high cost training environments [2]. 

Various skin substitutes have been utilised to simulate skin and soft tissue in the context of training for both basic surgical skills and more complex plastic surgical skills.

Biological tissues are commonly used and possess advantages such as low cost, and relative availability, as well as having some disadvantages for suitability in surgical skills training. Reliable and large-scale material acquisition may be difficult for larger cohorts of students, and the quality of material may vary. Animal tissue can have different characteristics to human skin including thickness and elasticity which may also be impacted if frozen and thawed. Considerations should be given for degradation and storage of the materials, hygiene and reusability of equipment. Biological tissue may also pose ethical and religious conflicts for participants. Fruits such as oranges have been used for surgical practice, however these have been found to be a less preferred model in comparison to biological and synthetic substitutes [3]. There are several commercially available, non-biological skin pads that are alternatives, however they vary considerably in their composition and fidelity to normal skin. Native skin possesses several key qualities including defined anatomical layers, elasticity, and lines of tension, which change with age. Understanding these biological principles and how to incorporate them into surgical technique is a key part of surgical training. The correct incorporation of these basic principles of tissue handling minimises surgical complications due to tissue damage, as well as aiding in achieving the optimal cosmetic and functional outcome. Substitute materials must attempt to mimic the biomechanics of normal tissues in order for skills such as suturing, lesion excision, local flap reconstruction and skin grafting to be effectively learned by surgical trainees.

COVID-19 has precipitated the need for remote learning and online courses in order to deliver surgical skills training [4]. Low-cost, high-fidelity materials, which are deliverable to a student’s own learning environment are necessary in order to facilitate this [5]. The source of such materials should ideally also be sustainable with regards to long-term availability, in order to maintain the standard of teaching disseminated to trainees over time. The environmental impact of materials from production to waste is an important consideration in the current world setting. 

This study aims to compare commercially available skin substitutes of a variety of materials including their cost and availability, assess their fidelity, and determine the optimal material for simulated skin surgical skills training programmes, particularly suturing, excision, and local flap skill development. 

## 2. Methods

### 2.1. Skin substitutes 

**Figure 1:**
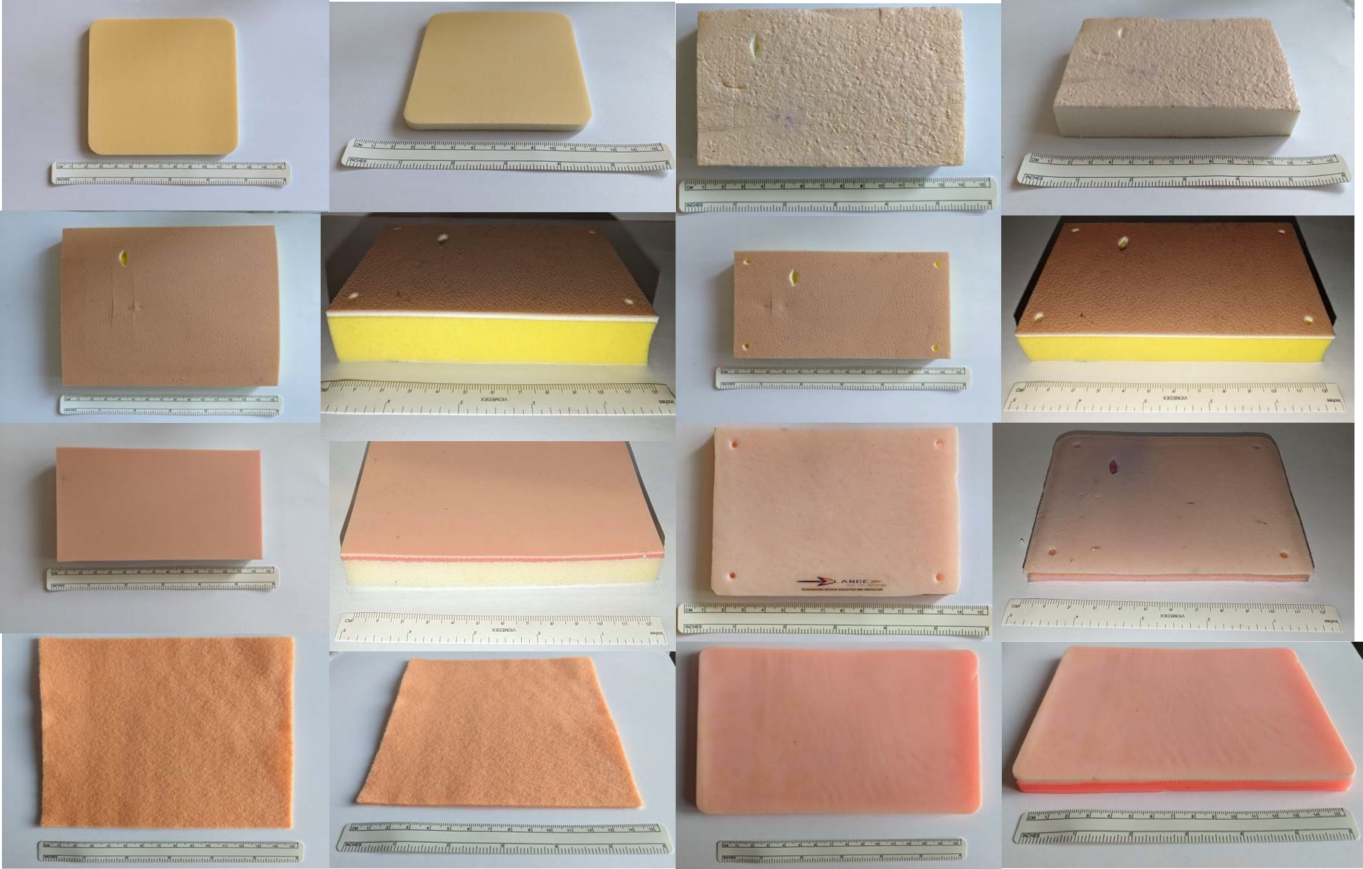
Skin Substitutes Reading left to right. Top Row (Row 1): Moderex foam dressing, Sigma Lance Basic. Row 2: Limbs and Things, Sigma Lance Advanced. Row 3: Suture Doctor, Sigma Lance Infinity. Bottom Row (Row 4): Felt, Generic Silicone.

Eight commercially available non-biological skin pads were chosen for inclusion in this study, covering a wide range of synthetic materials, representative of all available products. The skin pads were available through accessible online purchasing platforms such as Amazon^TM^ or a dedicated manufacturer website. These included Felt, Moderex non-adhesive foam polyurethane dressing, a generic silicone skin pad (GS), Suturing Doctor Skin (SD), Sigma Lance Basic (SLB), Sigma Lance Infinity (SLI) and Sigma Lance Advantage MK models (SLA) [6], [7], [8], [9], [10], [11], [12]. Limbs and Things MK skin pad was included in preliminary data extraction, however it was not included in the final survey due lack of availability to purchase at the time of the study (see figure 1 (Fig. 1)) [13]. Felt sheets are a well-known low cost, readily available craft material, and were included to determine their suitability for bench model simulation, in particular the properties which would assist training in suturing, excision and flap design such as elasticity, and manoeuvrability. The SLA model is used by the intercollegiate basic surgical skills course in the UK [10].

### 2.2. Outcome measures 

Data was collected for composition, defined layers, cost at the time of purchase, delivery time, subjective assessment by medical students, surgical trainees and surgeons, and objective measures of biomechanics including cutometry and durometry. Financial cost of each skin pad may vary over time, however, this criterion was included in this study due to the impacts upon logistical decision making and equipment requisition during course organisation. Cost of materials may vary in their market value, however, the financial cost of each material should be considered relative to each other at time of purchase. 

Delivery time may vary, however, large commercial retailers have standardised expectations for estimated delivery, which is generally consistent, and contributes to logistical planning. 

Cutometry provides a measure of tissue elasticity, whilst durometry measures the hardness of skin. These techniques provide quantifiable assessment and allow comparison of the mechanical properties both of biological and synthetic materials and have been widely utilised in assessing and monitoring of skin biomechanics in cutaneous diseases such as scleroderma [14], [15]. A cutometer produces a negative pressure, and once turned off, the skin will return to its previous state; this can measure firmness and elasticity of tissue [16]. A durometer attempts to make an indentation in the skin and measures the resistance of the material in order to assess hardness. These properties significantly contribute to direct closure of wounds and reconstruction of soft tissue defects, particularly in specialist areas such as the face, limbs and hands. A skin substitute which reflects these properties, will equip students with a greater understanding of tissue handling, recruitment of laxity and the properties of human skin in the context of surgical application. 

Cutometry and durometry data was collected for all skin substitutes and from three healthy non-smoking adults aged less than 30 years, with no medical co-morbidities. Measurements were collected from the forearm, forehead and back to compare the biomechanical properties of synthetic products to that of skin. Cutaneous properties of the face, back and arm demonstrate the most variation in consistency, thickness, elasticity, and durability. The measures were repeated three times per data point, and the scores averaged. These measures are representative of young healthy adults with no comorbidities however it is important to note they do not account for ageing skin or pathology which may affect these properties. 

A questionnaire developed by the authors was used to evaluate the skin pads application to surgical skills. The questionnaire consisting of eight criteria based upon task specific objectives, graded using a 1-5 Likert scale, was distributed amongst medical students, surgical trainees and consultants during the period of July 2021 until November 2021 (see attachment 1 ). Participants were asked to inspect the skin pads, perform interrupted sutures, a simple skin excision with closure, and incision with undermining of a pre-designed skin flap. Following completion of the tasks, participants were then asked to grade the skin substitutes, in relation to its similarity to skin with one equating to the unsuitable skin substitute, and five equating to excellent comparison to skin.

### 2.3. Ethical statement

Ethical approval was not required for this study in accordance with national guidelines of the Health Research Authority in the United Kingdom (U.K.), following completion of a proforma [17].

### 2.4. Data synthesis 

Data was tabulated for all outcome measures and the mean, mode and standard deviations calculated. The Kruskal Wallis test was used to compare cutometry and durometry of the skin substitutes to cutometry and durometry of the skin on the arm, back and forehead. This test was performed using Prism Graph Pad statistical software (GraphPad Software Inc, California, USA) [18].

## 3. Results

A comparison of material characteristics is shown in table 1 (Tab. 1). All materials were purchased from online distributors and were received within three days or less. All the skin substitutes were damage free and suitable for the review to be undertaken. The GS, SD and Limbs and Things skin pads have three defined layers either by a change of material or colour. Table 1 (Tab. 1) shows that the cheapest materials at the time of purchase were felt and the foam dressing, with the Limbs & Things skin pad being the most expensive per cm^2^. Materials were composed predominantly of foam and silicone. The foams and silicone used within each of the skin pads encompassed a range of compositions and densities.

**Figure 2:**
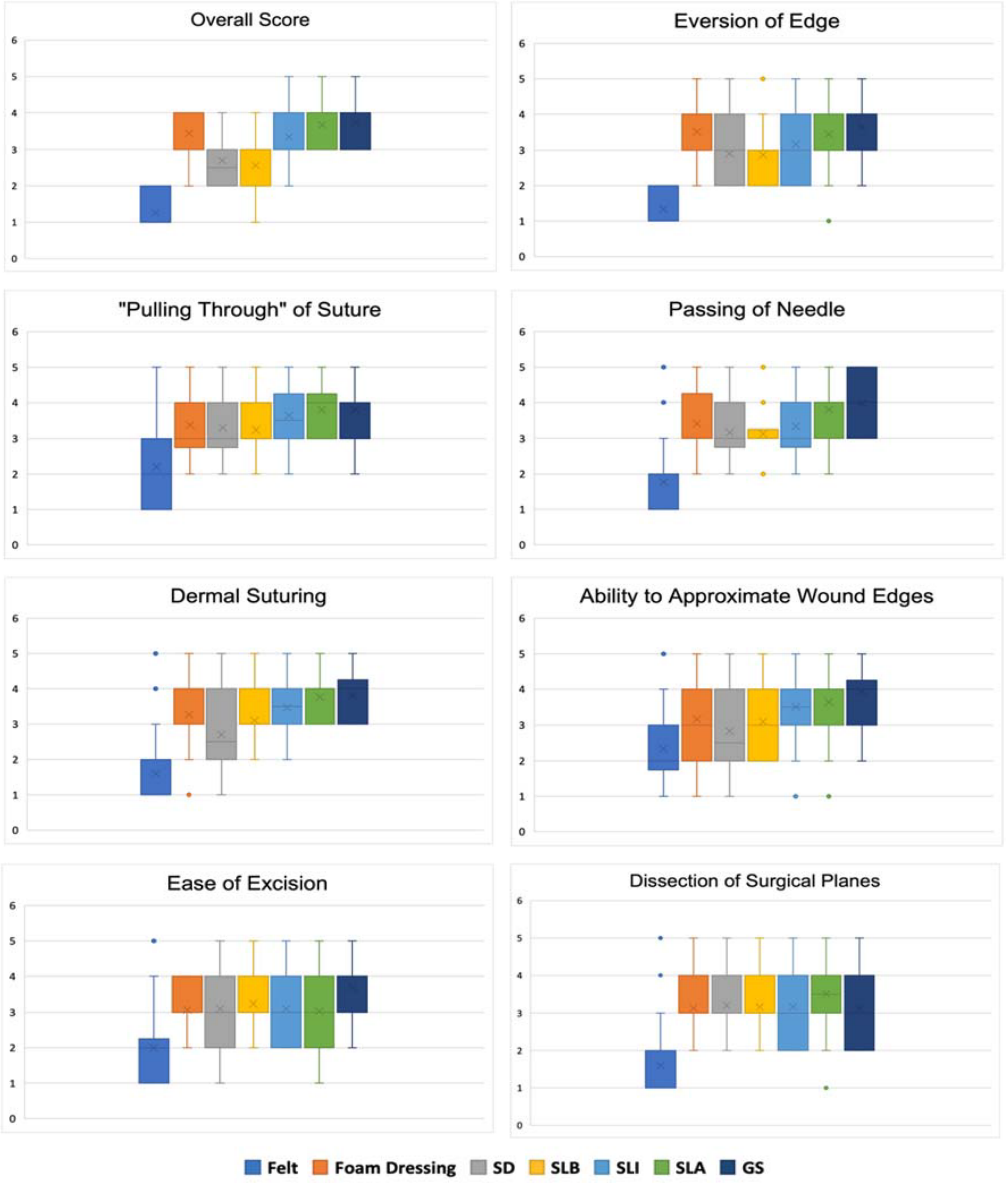
Surgical Skill Applications Questionnaire: Comparison of Skin Substitutes. Results are presented using box and whisker plots; mean, standard deviation, inter-quartile range and outliers are represented in each graph.

The questionnaire was completed by the entire sample of 30 trainees and practitioners: 15 medical students, eight surgical registrars and seven consultants within ENT and Plastic Surgery specialities. ENT and Plastic Surgery specialists were included as the skin measures (face, back and arm) and included surgical skills (suturing, excision, and local flap reconstruction) were relevant to their expertise. A comparison of each criterion is shown in figure 2 (Fig. 2). Overall, felt received the poorest score for suitability within surgical skills. Of the reviewed skin pads, the highest overall score was the GS substitute, scoring strongly against other materials within ease of excision. However, results showed that this skin pad had the highest range of scores for both dissection of surgical planes and passing of the needle through the material. There were no statistical differences in scoring between consultants/surgical trainees and medical students. 

Comparison of biomechanical properties showed that no significant statistical differences were identified between foam dressings and SLB when compared with skin of the face, arm and back in both cutometry and durometry. Overall, felt had consistently significant differences for both cutometry and durometry; felt was found only to be similar to cutometry data obtained from skin on the back (see table 2 (Tab. 2) and table 3 (Tab. 3)).

## 4. Discussion

Replication of real-life experiences through simulation training and incorporation of technological advances has become an integral part of medical education. Reproducible simulation learning environments are designed to teach students practical skills, as well as provide opportunities to develop communication, clinical prioritisation, and learn to collaborate with colleagues in high pressure environments, without the risks associated with workplace experiential learning [19]. 

Bench models are well known in the literature to produce improved skill acquisition. A randomised control trial by Grierson et al. demonstrated an improvement in the efficiency and manual dexterity of trainees performing an elliptical skin excision [20].

The development of a low-cost, high-quality plastic surgery skills course requires the provision of a skin substitute material capable of facilitating suturing, excision, and local flap training. The material needs to provide an easily distributable, cost-effective solution that was comparable to biological mechanical properties of skin. 

There is significant variance in the suitability of commercially available synthetic skin pads for surgical skill application. Cost of materials presents a practical barrier to wider access to skill development for trainees globally, particularly in low resource environments. Minimising expenses for students and educational bodies, without sacrificing the quality of training, is an important consideration especially with increased introduction of remote and online accessible training opportunities. 

Moderex non-adhesive foam dressings have shown to be the most cost-effective solution for the dissemination of a suitable product for surgical training in our study. This material received positive feedback from medical professionals of all experience levels, and comparable scoring when compared with skin substitutes specifically designed for surgical education, but at a much higher cost. Ease of manipulation, cost effectiveness and comparable rating amongst trainees highlight this as a potential candidate for surgical skill acquisition. Foam dressings can be easily replaced, following irreversible skill practice such as excision or local flap design, or can be re-used for suturing practice. This material is composed of two distinct foam layers, which allow for suturing practice, including dermal and subcutaneous techniques. Whilst felt was found the be the lowest costing material, it performed poorly in the survey, and was not found to be a close likeness to skin. Issues encountered included undefined layers, and cheese-wiring of sutures through the material.

Cutometry and durometry have been validated as methods of assessing skin’s mechanical properties [21]. These measurements of its physical qualities have been widely used in the literature to assess the degree of skin pathology such as fibrosis associated with systemic sclerosis, as well as monitoring the progression of disease and effectiveness of medical therapies [22]. There are significant differences in biomechanical properties of skin in various anatomical locations; the face can demonstrate a larger elastic potential than the back, whereas the back possesses a thicker, more rigid dermis [23]. Cutometry and durometry data demonstrated that the bilaminar foam material is closely comparable to skin biomechanically and reflects the potential application of this synthetic material to mimic skin in a simulation training environment.

Surgical trainees commented on the difficulty of practicing deep dermal sutures and tissue dissection techniques on all synthetic materials throughout this study. Materials were either not designed to replicate dermis and epidermis, or the material themselves were difficult to undermine and dissect through. Whilst several of the skin pads demonstrated a trilaminar structure, such as GS and SLI, the distinction was made through a change in colour, rather than a distinction in the material composition. Whilst appearance may assist in identification of correct surgical planes, dissection and separation of “tissues” was not comparable to skin. This continues to pose barriers to development in acquisition of local flap skills, and to a lesser extent simple skin excision. 

The SD skin pad was found to have a considerably more rigid material representation of the “dermis” in comparison to other substitute materials. This led to student difficulties in performing interrupted suturing, in particular eversion of the wound edge and passage of the needle through the materials. 

This study is limited to a comparison of synthetic substitutes to the skin of healthy volunteers below the age of 30. It is well known that the composition and biomechanical properties of skin can change with age and disease pathology and have demonstrated reduced elastic potential [24]. Further studies would be necessary to determine if there is a more suitable material that would correlate with ageing skin. 

## 5. Conclusion

Synthetic skin materials can be used to deliver surgical skill training for suturing, simple excision, and local flap design in a low-cost, high-fidelity manner. With the increasing use of remote learning and online education, synthetic skin substitutes can provide a cost effective, long lasting, reliable, and easily disseminated product to facilitate this. This paper has demonstrated that bilaminar foam polyurethane dressings provide a cheaper alternative to purposefully designed skin pads, whilst providing a comparable quality of simulation training. Tissue dissection remains a difficult skill to replicate in simulation training with skin substitutes and is a feature that products developed in future would benefit for developing further.

## Competing interests

The authors declare that they have no competing interests. 

## Supplementary Material

Questionnaire for comparison of skin substitutes

## Figures and Tables

**Table 1:**
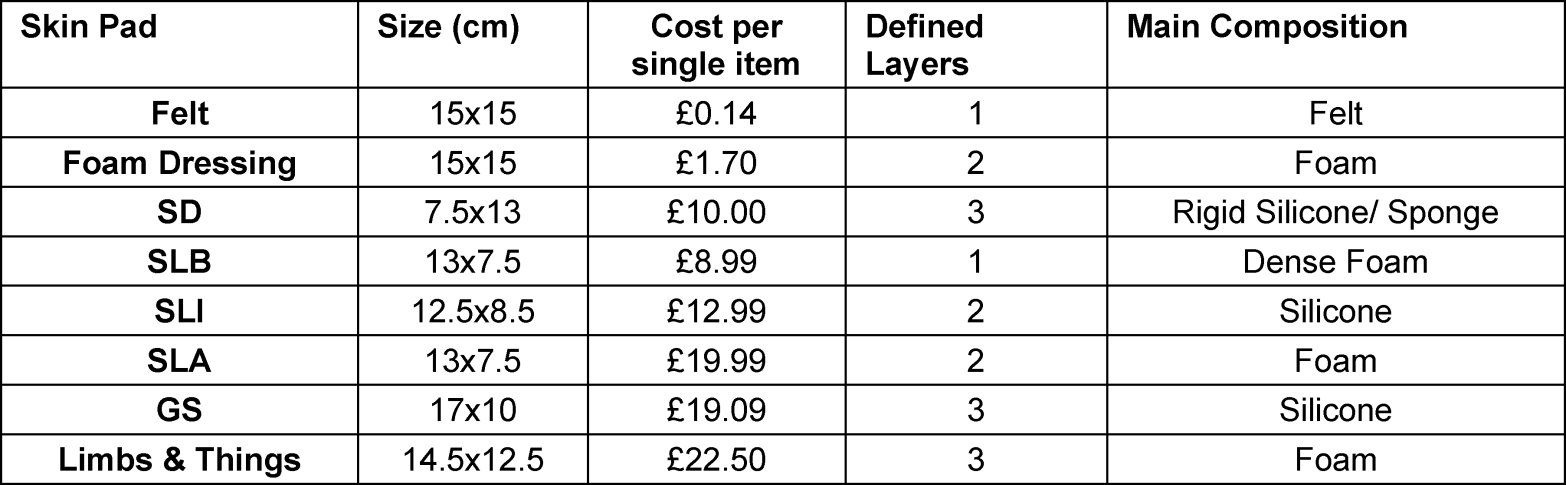
Comparison of Skin Substitute Characteristics SD=Suturing Doctor, SLB=Sigma Lance Basic, SLI=Sigma Lance Infinity, SLA=Sigma Lance Advanced, GS=Generic Silicone Felt (Model Number B093CDDJ53, manufactured by Rarco Ltd, London UK), Moderex non-adhesive foam dressing (Model Moderex PU foam dessing, manufactured by Zelador in Newport, South Wales), Generic silicone skin pad (Model Number 1911801MBZ, manufactured by Hemobllo, China) (GS), Suturing Doctor Skin Pad (Model Number SKU230624129, manufactured by Suturing Doctor, Bristol UK)(SD), Sigma Lance Basic (Model 137-HO-DO, manufactured by Sigma Lance in Surrey, UK) (SLB), Sigma Lance Infinity (Model Infinity MKI, manufactured by Sigma Lanxe in Surrey, UK) (SLI), Sigma Lance Advantage MK models (Model Advantage MKII, manufactured by Sigma Lance in Surrey, UK) (SLA), and Limbs and Things MK skin pad (Model 00092, manufactured by Limbs & Things in Bristol, UK)

**Table 2:**
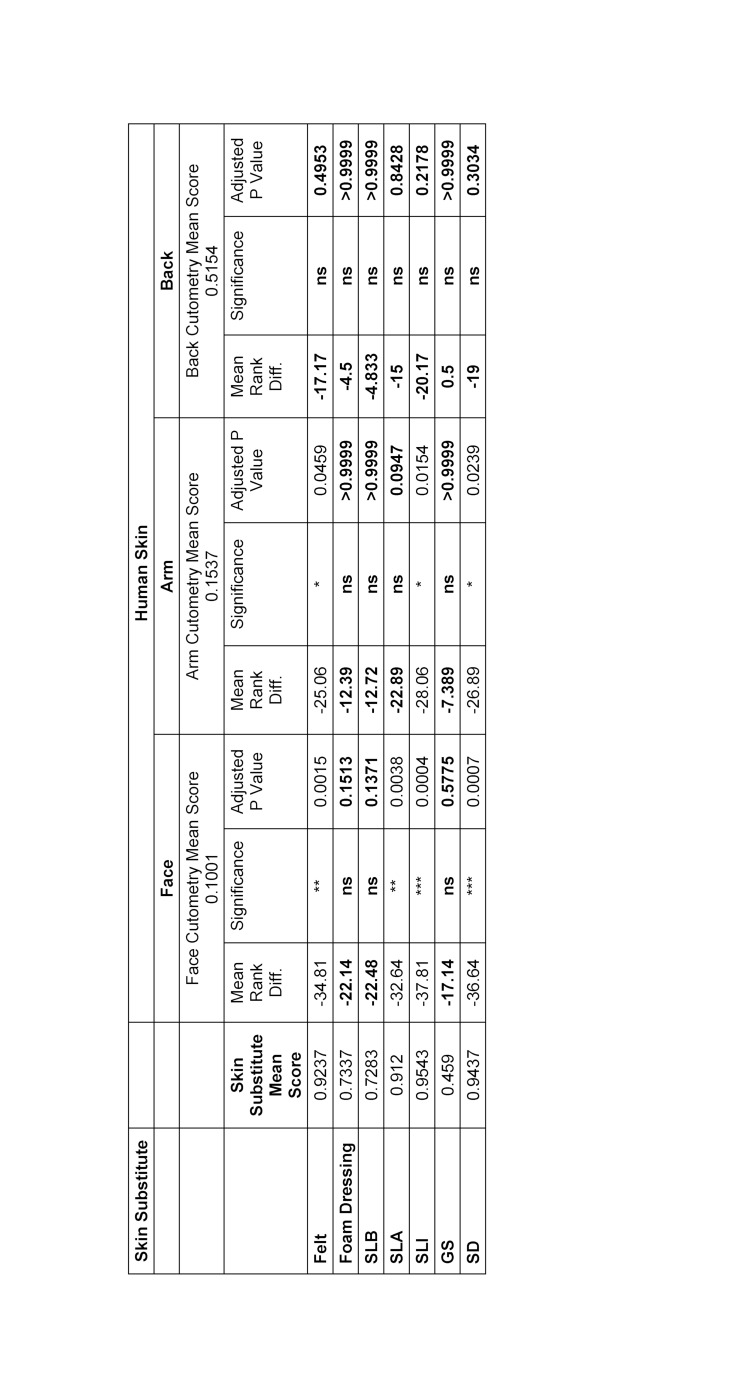
Comparson of Cutometry Outcomes of Skin Pads vs Skin using Kruskal Wallis Test SLB: Sigma Lance Basic, SLA: Sigma Lance Advantage, SLI: Sigma Lance Infinity, GS: Generic Silicone, SD: Suturing Doctor. Results highlighted in bold represents no significant difference in score between human skin and the substitute materials.

**Table 3:**
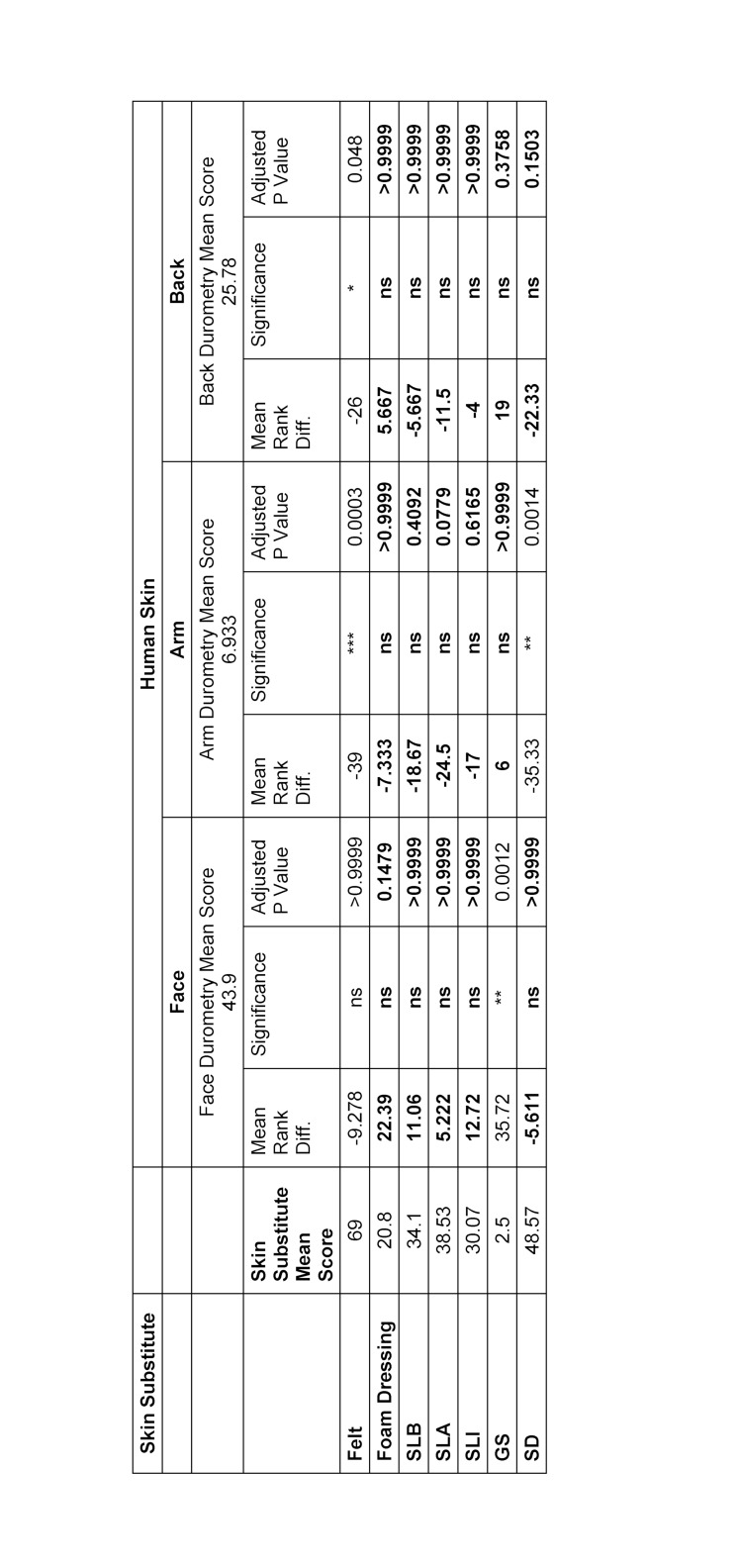
Comparson of Durometry Outcomes of Skin Pads vs Skin using Kruskal Wallis Test SLB: Sigma Lance Basic, SLA: Sigma Lance Advantage, SLI: Sigma Lance Infinity, GS: Generic Silicone, SD: Suturing Doctor. Results highlighted in bold represents no significant difference in score between human skin and the substitute materials.
