# Supplementary material for: A comparison of commercially available synthetic skin substitutes for surgical simulation training
Source: GMS J Med Educ. 2023 Sep 15;40(5):Doc62. doi: 10.3205/zma001644 (PMC10594032; doi:10.3205/zma001644)
Supplement: Questionnaire for comparison of skin substitutes [file JME-40-62-s-001.pdf]

Attachment 1: Questionnaire for comparison of skin substitutes

**Questionnaire Criteria**
Overall comparison to normal skin<br>Eversion of wound edge<br>Pulling through of the suture material<br>Passing of needle through the pad<br>Placement of a dermal suture<br>Ability to approximate edges
Ease of excision
Development of correct surgical planes<br><br>Grading<br>1 = unsuitable/unable to fullfill criteria<br>2= less than desired quality<br>3 = Average substitute for skin<br>4 = Good comparison to skin<br>5 = Excellent comparison to skin

Attachment 1 to Awad L, Langridge BJ, Jeon FH, Bollen E, Butler PE. *A comparison of commercially available synthetic skin substitutes for surgical simulation training.* GMS J Med Educ. 2023;40(5):Doc62. DOI: 10.3205/zma001644
